# Comparison of metagenomic next-generation sequencing and blood culture for diagnosis of bloodstream infections

**DOI:** 10.3389/fcimb.2024.1338861

**Published:** 2024-01-24

**Authors:** Juan Yu, Li Zhang, Deyu Gao, Jie Wang, Yi Li, Ning Sun

**Affiliations:** ^1^ Department of Clinical Laboratory, Nanjing Lishui People’s Hospital, Nanjing, China; ^2^ Department of Clinical Laboratory Science, Jinling Hospital, Affiliated Hospital of Medical School, Nanjing University, Nanjing, China; ^3^ Clinical Medicine Research Center, The Affiliated Suqian First People’s Hospital of Nanjing Medical University, Suqian, China

**Keywords:** bloodstream infections, plasma cell-free DNA, metagenomic next-generation sequencing, 16S rRNA, diagnosis

## Abstract

**Objectives:**

This study aimed to evaluate the clinical performance of plasma cell-free DNA (cfDNA) next-generation sequencing (NGS) for pathogen detection in patients with sepsis.

**Methods:**

A total of 43 pairs of blood and plasma samples form 33 blood culture-positive patients were used as testing samples in metagenomic NGS (mNGS) and NGS of 16S ribosomal RNA gene amplicons (16S rRNA NGS). The results of routine tests, including microbial culture, complete blood count, and biochemical tests, were collected from electronic medical records.

**Results:**

Using blood as an mNGS testing sample, the proportion of host DNA was 99.9%, with only three bacteria and no fungi detected. When using plasma in mNGS, the proportion of host DNA was approximately 97%, with 84 bacteria and two fungi detected. Notably, 16S rRNA NGS detected 15 and 16 bacteria in 43 pairs of blood and plasma samples, respectively. Blood culture detected 49 bacteria (23 gram-negative bacilli and 26 gram-positive cocci) and four fungi, with 14 bacteria considered contaminants by clinical microbiologists. For all blood cultures, plasma cfDNA mNGS detected 78.26% (19/23) gram-negative rods, 17% (2/12) gram-positive cocci, and no fungi. Compared to blood cultures, the sensitivity and specificity of plasma cfDNA mNGS for detecting bacteria and fungi were 62.07% and 57.14%, respectively.

**Conclusion:**

Compared to blood, plasma is more suitable for the detection of bloodstream infections using mNGS and is less affected by host DNA. The positive detection rate of plasma cfDNA mNGS for bloodstream infections caused by gram-negative bacteria was higher than that caused by gram-positive cocci.

## 1 Introduction

Bloodstream infection (BSI) is a serious disease caused by various pathogens, such as bacteria, fungi, and viruses, which enter and reproduce in the bloodstream (Martinez and Wolk, 2016; Lamy et al., 2020). It usually occurs in compromised patients, such as those in intensive care units or those with long-term hospital stays, organ transplant recipients, and patients with tumors, and often lead to severe complications, such as sepsis, shock, and even death (Martinez and Wolk, 2016; Lamy et al., 2020). The sources of BSIs are diverse and include surgical incision, lung, catheter-related, and abdominal infection. An early and accurate diagnosis is crucial for timely and appropriate treatment.

BSIs can be diagnosed based on clinical symptoms, blood biochemical markers, blood cultures, and nucleic acid amplification tests (Martinez and Wolk, 2016; Gu et al., 2019; Lamy et al., 2020; Gu et al., 2021; Yan et al., 2023). Blood culture is the most common method for the detection and identification of bacteria and fungi in patients with sepsis and can also optimize the use of antimicrobial drugs and evaluate treatment effectiveness (Garcia et al., 2015; Opota et al., 2015; Fabre et al., 2022). However, the turnaround time for blood cultures is usually over 24 hours and is prone to false positives and negatives (Garcia et al., 2015). Recently, metagenomic next-generation sequencing (mNGS) has attracted widespread attention for use in pathogen detection (Gu et al., 2019; Gu et al., 2021; Yan et al., 2021). It is a culture-independent detection method that can detect all pathogens and identify rare or unknown pathogens. However, mNGS also has disadvantages, such as complex sample preparation, cumbersome sequencing procedures, and the effect of host DNA. Several studies have shown that plasma cell-free DNA (cfDNA) mNGS (plasma mNGS) has significant value in the BSI detection and can significantly reduce the adverse effects of host DNA (Grumaz et al., 2016; Blauwkamp et al., 2019; Barrett et al., 2020; Yan et al., 2021; Park et al., 2023; Xu et al., 2023). cfDNA in the plasma is fragmented DNA derived from dead microorganisms, whereas whole blood samples may contain live pathogens and higher concentrations of microbial genomic DNA. Direct evidence is needed to confirm that detecting cfDNA in the plasma using mNGS is a good choice for clinical performance. Additionally, BSIs can also be identified using the NGS-targeted 16S rRNA gene (16S rRNA NGS), which can not only alleviate the impact of host DNA, but also significantly reduce the cost of testing (Rutanga et al., 2018). Similar to mNGS, the detection and identification of BSIs using 16S rRNA NGS can be performed directly on whole blood or plasma samples (El Gawhary et al., 2016; Lelouvier et al., 2016; Moore et al., 2016; Païssé et al., 2016; Fida et al., 2021). More data are required to compare and evaluate the clinical performance of 16S rRNA NGS in the diagnosis of BSIs.

In this study, we aimed to evaluate the clinical performance of 16S rRNA NGS and mNGS (plasma mNGS, blood mNGS, plasma 16S RNA NGS, and blood 16S RNA NGS) in the detection and identification of pathogens in patients with sepsis using plasma and blood as test samples.

## 2 Materials and methods

### 2.1 Study overviews and subjects

This is a non-interventional and retrospective study. Between April 2023 and June 2023, 198 residual peripheral venous blood samples were collected after routine testing from 170 patients who underwent blood culture testing on the same day. Samples with positive blood cultures were retained for mNGS and 16S rRNA NGS, whereas those with negative blood cultures were excluded. The sources of BSIs were determined based on microbiological culture, clinical symptoms, imaging examination, and treatment response. Any remaining blood samples were stored in standard EDTA tubes, with an approximate volume of 2 ml. One microliter of peripheral venous blood samples was immediately stored at -80°. The remaining samples were centrifuged at 16000×g for 10 min, and 800 μl of plasma was collected and stored at -80°. This study was approved by the Ethics Committee of Jinling Hospital and People’s Hospital of Lishui (Nanjing, China), and informed consent was not required because patient information was anonymized and residual samples were obtained after routine testing.

### 2.2 Blood cultures

In our clinical microbiology laboratory, the standard blood culture involved two sets: one set included a pair of BD BACTEC Plus Aerobic/F bottles and BD BACTEC Lytic Anaerobic/F bottles (Becton Dickinson, Heidelberg, Germany), and the other set consisted of a pair of aerobic and anaerobic blood culture bottles (BacT/ALERT FA/FN Plus, bioMérieux, Marcy-l’Etoile, France). The blood culture bottles were incubated for 5 days. Cultures from positive blood culture bottles were inoculated onto blood, chocolate, and MacConkey agars, followed by microscopic testing. Biochemical identification was performed using the VITEK2 COMPACT system (bioMérieux, Marcy-l’Etoile, France).

### 2.3 Nucleic acid extraction

Nucleic acid was extracted from 600 μl of peripheral venous blood and plasma using a modified RNA/DNA Purification Kit (Magnetic Bead) in a Stream SP96 Automatic Nucleic Acid Extraction System (DaAnGene, Guangzhou, China). Finally, nucleic acid was eluted in 50 μl of sterile deionized water.

### 2.4 Metagenomic NGS

Reverse transcription of RNA was performed using PrimeScript™ 1st Strand cDNA Synthesis Kit (Takara, Dalian, China). Subsequently, double-stranded cDNA was synthesized using RNase H and DNA Polymerase I (Takara, Dalian, China) and purified using TaKaRa MiniBEST DNA Fragment Purification Kit Ver.4.0 (Takara, Dalian, China). Double-stranded cDNA was mixed with equal volume of nucleic acid elution. DNA was randomly fragmented using Covaris S220 (Covaris, Woburn, MA, USA) to an average size of 300−350 bp. The fragmented DNA was quantified using a Qubit@ 2.0 Fluorometer (Thermo Fisher Scientific Inc., USA). Library preparation, including end repair, adaptor linking, purification, and PCR amplification, was performed using the VAHTS Universal Pro DNA Library Prep Kit for Illumina (Vazyme Biotech Co., Ltd., Nanjing, China). The library was determined and quantified by Agilent 2100 Bioanalyzer and Qubit@ 2.0 Fluorometer (Thermo Fisher Scientific Inc., USA). NGS was performed using the NovaSeq platform (Illumina, San Diego, CA, US) with 2×150 paired ends.

### 2.5 16S rRNA NGS

As described in our previous study (Sun et al., 2023), the V3−V4 region of the 16S rRNA gene was amplified using two universal primers with adaptor (341F and 806R), and NGS was performed using a NovaSeq platform (Illumina, San Diego, CA, US) with 2×250 paired ends.

### 2.6 Negative and internal controls

Sterile deionized water and three paired samples of peripheral venous blood and plasma collected from healthy individuals after routine testing were used as the blank and negative controls, respectively. Artificial synthetic DNA (7.5×10^4 copies) was used as an internal control and spiked into all samples and controls to serve as a quality control. All controls were processed in parallel with all the samples.

### 2.7 Bioinformatic analysis

Raw data obtained from mNGS were analyzed using a previously-described protocol (Lu et al., 2022), and data obtained from 16S rRNA NGS were analyzed using the Basic Local Alignment Search Tool (BLAST, version 2.12.0+, https://blast.ncbi.nlm.nih.gov/Blast.cgi) (Rognes et al., 2016). Low-quality mNGS and 16S rRNA NGS data were removed using Fastp software (Chen et al., 2018). Host DNA reads were removed after alignment with the human reference genome GRCh38 using BWA-MEM (Li, 2013). The clean data were annotated with a prebuilt database (https://genome-idx.s3.amazonaws.com/kraken/k2_standard_eupath_ 20201202.tar.gz) using kraken2 software. The clean reads from16S rRNA NGS were clustered using VSEARCH with 100% identity (Rognes et al., 2016). Operational taxonomic units were annotated using BLAST+ against the National Center for Biotechnology Information 16S rRNA database.

### 2.8 Criteria for pathogen identification

The criteria for pathogen identification were developed as described in the previous studies (Blauwkamp et al., 2019; Gu et al., 2019; Barrett et al., 2020; Gu et al., 2021; Wang et al., 2021; Yan et al., 2021; Xu et al., 2023). For mNGS, the reads per million (RPM) and Z-scores of each species per sample were calculated using Pavian (https://fbreitwieser.shinyapps.io/pavian/) and compared with negative controls. Because of the concentration of host DNA, we compared the mNGS results with the corresponding sample types of negative controls, for example, plasma samples versus plasma negative controls. The criteria were as follows: 1) The Z-score of pathogens in the sample was three-fold higher than that in negative controls; 2) the reads were strictly mapped to three different regions of the genome; and 3) the species with the highest abundance were retained when the RPM was more than five times. Furthermore, each species was considered a pathogen when it was considered clinically relevant by clinicians or had been reported in the literature. The detection and identification of pathogens using 16S rRNA NGS was based on our previous study (Sun et al., 2023).

### 2.9 Statistical analysis

Statistical analysis was performed using R software (Version 4.3.0). Continuous variables are presented as means and standard deviations, and categorical variables are presented as counts and percentages. The results of blood and plasma mNGS were compared using the non-parametric Mann-Whitney U test. Clinical sensitivity and specificity were calculated using standard formulas and evaluated using Fisher’s exact test.

## 3 Results

### 3.1 Patient characteristics

Of the 198 samples, 43 blood culture-positive samples collected from 33 hospitalized patients were screened to evaluate the clinical performance of NGS in the detection and identification of pathogens. The mean age was 50 years old, and 76% (25/33) of patients were men. The patients included in the study presented with signs and symptoms of infection, including respiratory and abdominal infections. They were promptly treated with empirical anti-microbial therapies, such as piperacillin/tazobactam or biapenem. Blood culture testing was administered when their body temperature exceeded 38°. The mean length of the hospital stay was 31 days. The median white blood cell count was 11.7 × 10^9 cells/l, median lymphocyte count was 0.67 × 10^9 cells/l, and median neutrophils were 9.6 × 10^9 cells/l. The median values of C-reactive protein and procalcitonin were 91.6 mg/l and 1.525 μg/l, respectively, with the levels exceeding the normal range. The hospitalization diagnoses included three cases of intestinal fistula, one case of abdominal injury, two cases of lung infection, one case of abdominal pain, one case of rapidly progressive nephritis, 20 cases of acute severe pancreatitis, one case of Crohn’s disease, one case of chronic renal insufficiency, one case of duodenal fistula, one case of gastrointestinal bleeding, and one case of tremor.

### 3.2 Comparison of blood and plasma mNGS in the detection of pathogen

**Figure 1:**
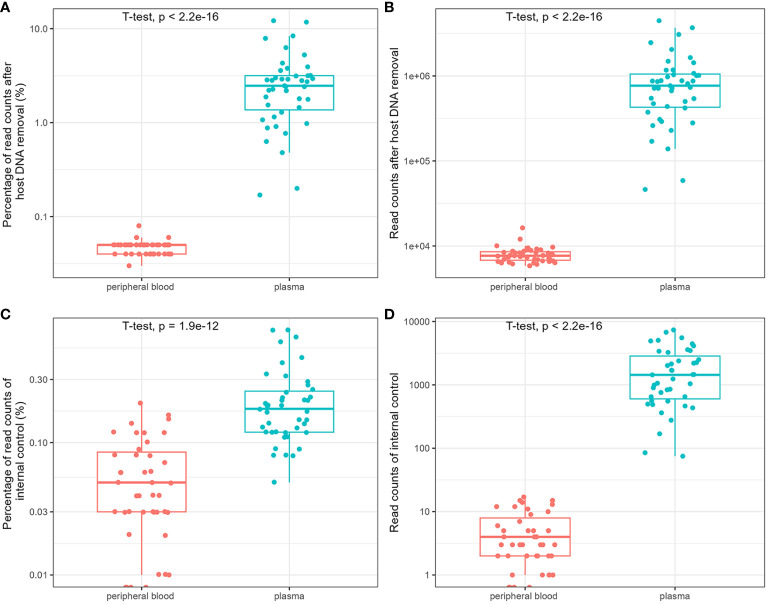
Comparison of the influence of host DNA on detection and identification of pathogens using metagenomic next-generation sequencing (mNGS). The relative abundance and reads of non-host DNA **(A, B)** and internal controls **(C, D)**.

The influence of host DNA on the detection and identification of pathogens using mNGS was analyzed using DNA extracted from the blood and plasma. After removing host DNA reads, the mean number of reads of plasma mNGS accounted for 3.04% (95% CI: 2.21% to 3.86%; reads: 9.83 × 10^5), whereas the mean number of reads of blood mNGS is 0.05% (95% CI: 0.045% to 0.050%; reads: 7.96 × 10^3). There was a significant difference between the two groups (**Figures 1A, B**). We compared the reads of the internal controls spiked with plasma and blood samples. The results showed that no reads were determined in the three blood samples, and the mean number of reads in all blood samples were five (relative abundance was 0.059%; 95% CI: 0.044% to 0.073%). For plasma mNGS, the mean number of reads are 2 × 10^3 (relative abundance of 0.22%; 95% CI: 0.17% to 0.27%). A significant difference was observed in the detection of internal controls between the two methods (**Figures 1C, D**). Therefore, plasma is a better choice for the detection and identification of pathogens in BSIs using mNGS.

### 3.3 Comparison of mNGS and 16S rRNA NGS using blood and plasma as test samples

**Figure 2:**
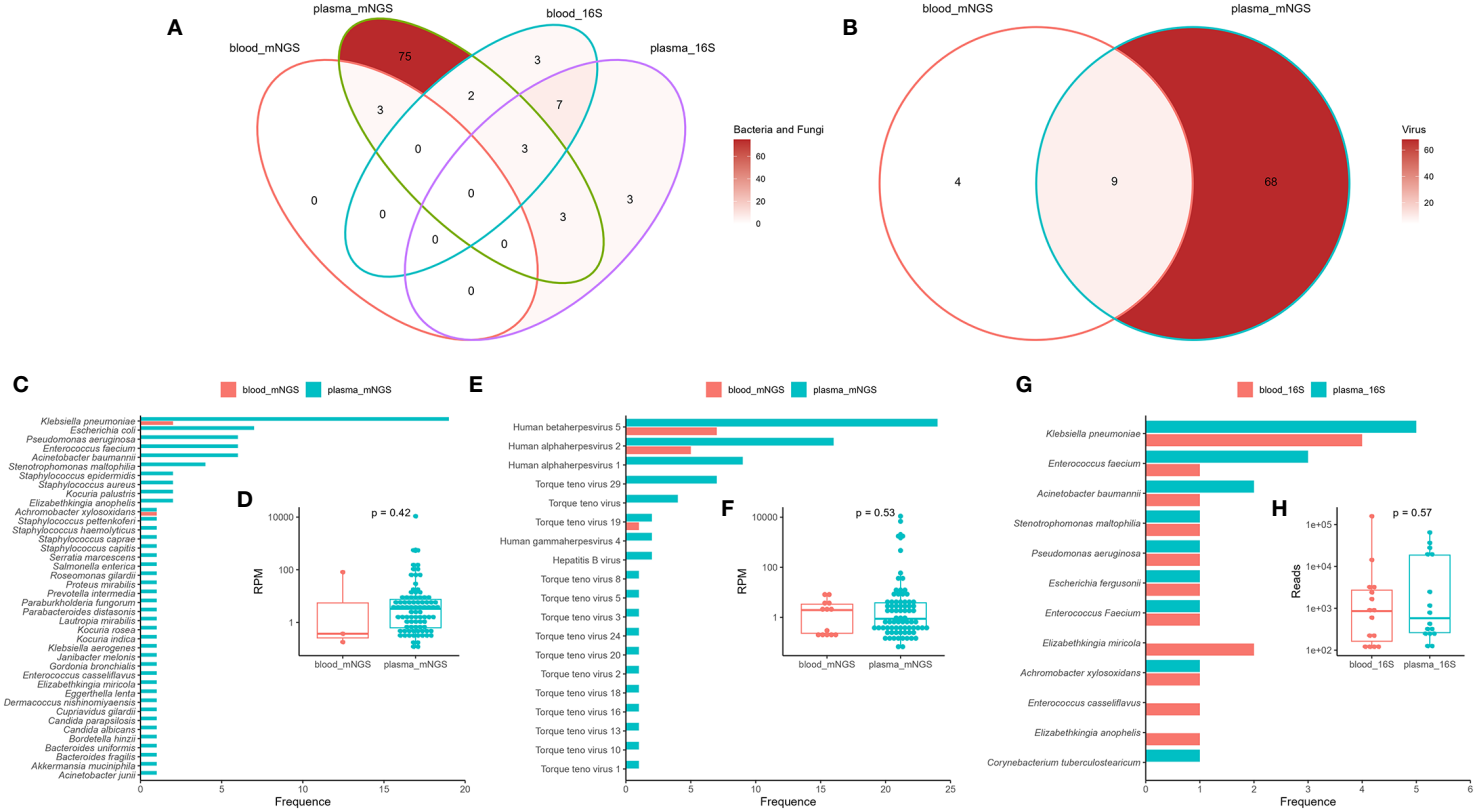
Comparison of mNGS and 16S rRNA gene next-generation sequencing (16S rRNA NGS) in detection of bloodstream infections using blood and plasma as test samples. Venn diagram analysis of the consistency of the detection results (**A**: bacteria and fungi; **B**: viruses) using mNGS and 16S rRNA NGS. Distribution and reads per million (RPM) of microbes (**C**, **D**: bacteria and fungi; **E**, **F**: virus) detected by mNGS. Distribution **(G)** and reads **(H)** of bacteria detected by 16S rRNA NGS. Plasma_mNGS, mNGS using plasma as a test sample. Blood_mNGS, mNGS using blood as a test sample. Plasma_16S, 16S rRNA NGS using plasma as a test sample. Blood_16S, 16S rRNA NGS using blood as a test sample.

Blood mNGS detected only three bacteria in there samples (sample ID: B02, B12, and B43), which were also detected by plasma mNGS (**Figure 2A**). However, plasma mNGS detected 84 bacteria belonging to 26 genera and 40 species, as well as two fungi (**Figure 2A**), of which 22% (19/86) were *Klebsiella pneumoniae* (**Figure 2C**). The median RPM of bacteria and fungi were 3.29 (range: 0.11 to 10801.45; **Figure 2D**). Notably, various DNA viruses were detected (**Figure 2E**), such as human betaherpesvirus 5 (CMV), human alphaherpesvirus 1 (HSV1), human alphaherpesvirus 2 (HSV2), torque teno virus (TTV), human gammaherpesvirus (EBV), and hepatitis B virus (HBV), and the median RPM was 0.91 (range: 0.07 to 10958.91; **Figure 2F**). No RNA viruses were detected using mNGS. The types and abundance of DNA viruses detected in the plasma were higher than those in the blood samples (**Figures 2B, E**). The results of 16S rRNA NGS for detecting bacteria in the blood and plasma samples showed that there were only 10 consistent bacteria between the two methods (**Figure 2A**), and there was no significant difference in read counts (**Figures 2G, H**). By comparing mNGS and 16S rRNA NGS, we found that plasma mNGS could detect more potential pathogens.

### 3.4 Comparison of plasma cell-free mNGS with blood cultures

**Figure 3:**
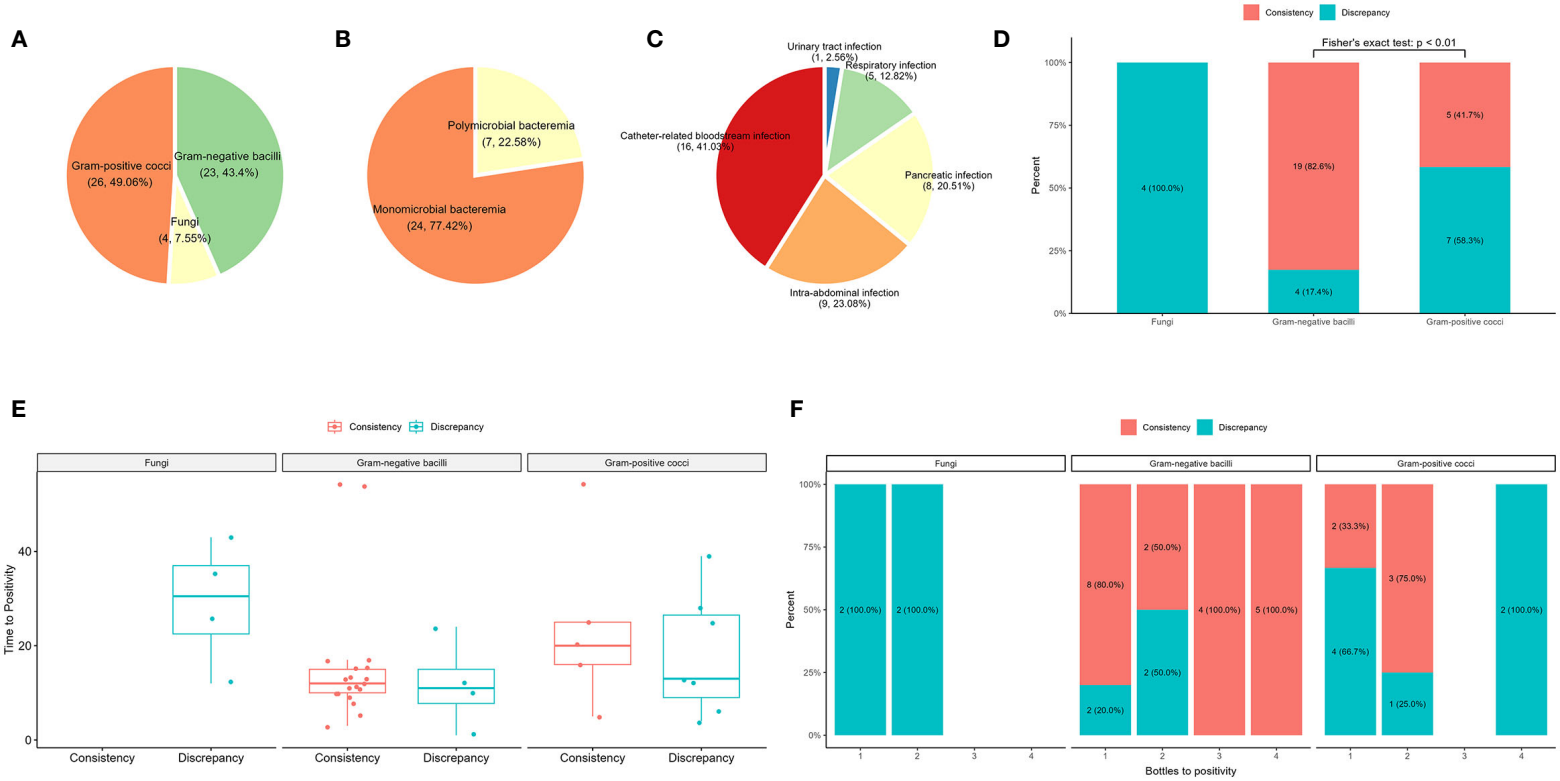
Comparison of plasma mNGS and blood culture in the detection of bacteria and fungi. **(A)** Distribution of blood culture. **(B)** Composite of monomicrobial and polymicrobial bacteremia after removal of the contaminating strains. **(C)** The source of bloodstream infections. **(D)** The influence of the type of pathogen detected by plasma mNGS versus blood culture. **(E)** Effect of time of positivity of blood culture on plasma mNGS. **(F)** Number of bottles of blood culture used in plasma mNGS.

Of the 43 samples, blood culture detected 49 bacteria (23 gram-negative bacilli and 26 gram-positive cocci) and four fungi, and 14 gram-positive cocci were determined to be contaminants by clinical microbiologists (**Figure 3A**; **Supplementary Table S1**). After removing the contaminating strains, 31 blood culture-positive samples (72%) were obtained, with 24 samples showing bacteremia caused by a single bacterium and seven samples containing two or more pathogens (**Figure 3B**). Analysis of the source of BSIs showed that 16 were catheter-related infections, nine were abdominal infections, eight were pancreatic infections, five were respiratory tract infections, and one was a urinary tract infection (**Figure 3C**). The comparison between plasma mNGS and blood culture showed a consistency rate of 78.26% (19/23) for gram-negative bacteria and only 17% (2/12) for gram-positive cocci (**Figure 3D**). Both fungi were detected by blood culture, whereas the 14 gram-positive contaminants were not detected by plasma mNGS. The mean time of positivity to detect gram-positive bacteria was 19.4 hours, which was longer than the mean time of positivity to detect gram-negative bacteria (15.1 hours); however, there was no significant difference (p = 0.473, **Figure 3E**). There was no significant correlation between the number of positive blood culture bottles and plasma mNGS results (**Figure 3F**). The type of bacteria had a significant influence on plasma mNGS, with higher positivity for gram-negative bacteria than for gram-positive bacteria and fungi.

The plasma mNGS results were consistent with the blood culture results for 26 samples, including 18 blood culture-positive samples and eight false-positive samples (**Table 1**, **Supplementary Table S1**). However, there were differences between the blood culture and plasma mNGS results for 17 samples (**Table 2**). Thirteen samples were positive for blood culture, whereas four samples were false positives. The source of BSIs in the 13 samples from 11 patients included seven catheter-related BSIs, two respiratory tract infections, three pancreatic infections, and one intra-abdominal infection. In sample B10, *Enterococcus faecium* was not detected by plasma mNGS; however, consecutive blood and catheter tip cultures indicated catheter-related BSIs. In sample B12, *Candida auris* was not detected by plasma mNGS; however, infection source analysis showed that the patient had respiratory tract-, pancreatic-, and catheter-related infections. Case P17 (samples B19 and B20) involved pancreatic infection and catheter-related BSI, with catheter tip culture results showing *Enterococcus faecalis* and *C. parapsilosis*, and pancreatic fluid culture showing *K. pneumoniae* and *E. faecalis*. *Acinetobacter baumannii* was not detected in two samples by plasma mNGS. In case P03 (sample B03), the aerobic bottle in the lower left quadrant of the blood culture showed a positive result with a positivity time of 10 hours. The other three bottles were negative after 5 days of incubation. The patient’s pancreatic fluid culture revealed *K. pneumoniae*, which was also detected by plasma mNGS. In case P18 (sample B21), blood cultures revealed *A. baumannii* by blood culture, whereas plasma mNGS detected *Acinetobacter junii*. *Staphylococcus aureus* was not detected in two cases. Patient P06 had glomerulonephritis and developed a catheter-related BSI. However, the culture results of the catheter tip, pus, and blood revealed *S. aureus*. Plasma mNGS detected *Kocuria rosea* and *Janibacter melonis*, which are skin surface colonizers and potential pathogens that cause BSIs. In case P24 (samples B25 and B28, blood cultures 24 hours interview), *Staphylococcus hominis*, a common skin surface commensal and an unusual pathogen causing catheter-related BSI, was detected, whereas plasma mNGS detected *K. pneumoniae* and *E. faecium*, which were also detected by pancreatic fluid culture. Case P11 (sample B13) presented with a gastrointestinal hemorrhage. During hospitalization, respiratory tract infection and catheter-related BSI were observed. *Candida parapsilosis* was cultured from aerobic bottles for 12 hours on both sides. Plasma mNGS detected *Pseudomonas aeruginosa*, which was consistent with the culture of sputum and bronchoalveolar lavage before 24 hours. In Case P21 (sample B25), *Burkholderia cepacia* was detected by blood culture, with all four bottles showing positive results after 24 hours. However, plasma mNGS only detected *Staphylococcus epidermidis* and *Elizabethkingia miricola*, which were also detected by plasma 16S rRNA NGS. Case P09 (B33) had a catheter-related BSI. *E. faecalis* and *Stenotrophomonas maltophilia* were cultured from the blood culture after 2 days and from catheter tip culture after 1day. Notably, the plasma mNGS did not detect any pathogens on the same day. However, both *E. faecalis* and *S. maltophilia* were detected by plasma mNGS after 2 days.

**Table 1:** Concordant results between blood culture and plasma cell-free DNA mNGS.

Patient ID	sample ID	Gender	Disease^a^	Age	Admission date	Discharge date	Sampling date	Bottle positives	Time to positivity	Results of blood culture	Results of plasma mNGS	Source of blood culture
P01	B01	male	SAP	37	2023/3/9	2023/4/30	2023/4/25	1/4	10	*Acinetobacter baumannii*	*Staphylococcus aureus*, *Klebsiella pneumoniae*, *Acinetobacter baumannii*	Catheter-related bloodstream infection
P02	B02	male	SAP	47	2023/4/21	2023/4/28	2023/4/26	4/4, 2/4	11, 25	*Klebsiella pneumoniae, Enterococcus faecium*	*Klebsiella pneumoniae*, *Acinetobacter baumannii*, *Enterococcus faecium*	Intra-abdominal infection
P04	B04	male	SAP	53	2023/3/24	2023/5/11	2023/4/27	4/4	13	*Pseudomonas aeruginosa*	*Pseudomonas aeruginosa*, Human alphaherpesvirus 1, *Staphylococcus haemolyticus*, Human betaherpesvirus 5	Catheter-related bloodstream infection
P04	B09	male	SAP	53	2023/3/24	2023/5/11	2023/5/6	1/4	17	*Pseudomonas aeruginosa*	*Pseudomonas aeruginosa*, *Stenotrophomonas maltophilia*, Human betaherpesvirus 5, Human alphaherpesvirus 1	Catheter-related bloodstream infection
P05	B05	female	AP	50	2023/4/10	2023/5/10	2023/5/3	3/4, 1/4, 3/4	9, 13, 15	*Pseudomonas aeruginosa, Klebsiella pneumoniae, Stenotrophomonas maltophilia*	*Pseudomonas aeruginosa*, *Stenotrophomonas maltophilia*, *Klebsiella pneumoniae*, Human betaherpesvirus 5	Catheter-related bloodstream infection
P08	B08	male	Pulmonary infection	87	2023/4/18	2023/6/2	2023/5/5	1/4	13	*Klebsiella pneumoniae*	Human betaherpesvirus 5, *Klebsiella pneumoniae*	Urinary tract infection
P10	B11	female	intestinal fistula	48	2023/4/26	2023/5/15	2023/5/6	3/4, 1/4	3, 54	*Klebsiella pneumoniae, Escherichia coli*	*Klebsiella pneumoniae*, *Pseudomonas aeruginosa*, *Bacteroides uniformis*, *Parabacteroides distasonis*, *Akkermansia muciniphila*, *Eggerthella lenta*, *Escherichia coli*, Human betaherpesvirus 5	Intra-abdominal infection
P12	B14	male	abdominal pain	53	2023/5/10	2023/5/11	2023/5/10	4/4	12	*Escherichia coli*	*Escherichia coli*, *Enterococcus casseliflavus*, Human betaherpesvirus 5	Intra-abdominal infection
P13	B15	male	pulmonary infection	35	2023/5/10	2023/5/18	2023/5/11	1/4	15	*Klebsiella pneumoniae*	*Klebsiella pneumoniae*, *Dermacoccus nishinomiyaensis*, *Acinetobacter baumannii*, Human betaherpesvirus 5	Respiratory infection
P13	B22	male	pulmonary infection	35	2023/5/10	2023/5/18	2023/5/11	1/4	15	*Acinetobacter baumannii*	*Klebsiella pneumoniae*, *Acinetobacter baumannii*	Respiratory infection
P14	B16	male	AP	43	2023/4/3	2023/5/20	2023/5/11	1/4	17	*Klebsiella pneumoniae*	*Klebsiella pneumoniae*, Human betaherpesvirus 5	Pancreatic infection
P15	B17	male	SAP	55	2023/5/4	2023/5/22	2023/5/11	2/4	11	*Acinetobacter baumannii*	*Escherichia coli*, *Klebsiella pneumoniae*, *Cupriavidus gilardii*, *Salmonella enterica*, *Acinetobacter baumannii, Pantoea ananatis*, Human betaherpesvirus 5	Intra-abdominal infection
P15	B35	male	SAP	55	2023/5/4	2023/5/22	2023/5/11	2/4	11	*Enterococcus faecium*	*Elizabethkingia anophelis*, *Escherichia coli*, *Enterococcus faecium*	Intra-abdominal infection
P16	B18	male	SAP	38	2023/5/12	2023/6/8	2023/5/12	1/4	11	*Klebsiella pneumoniae*	*Klebsiella pneumoniae*	Pancreatic infection
P19	B23	female	AP	50	2023/3/20	2023/5/18	2023/5/16	1/4	12	*Acinetobacter baumannii*	*Staphylococcus aureus*, *Escherichia coli*, *Proteus mirabilis*, *Acinetobacter baumannii*	Catheter-related bloodstream infection
P20	B24	male	duodenal fistula	53	2023/5/15	2023/5/26	2023/5/17	3/4	5	*Klebsiella pneumoniae*	*Klebsiella pneumoniae*, *Escherichia coli*, Human betaherpesvirus 5	Intra-abdominal infection
P25	B29	female	AP	66	2023/4/26	2023/6/10	2023/4/27	2/4	16	*Enterococcus faecium*	*Enterococcus faecium*	Pancreatic infection
P28	B36	female	psychogenic tremor	88	2023/5/9	2023/5/16	2023/5/6	2/4	12, 23	*Staphylococcus epidermidis*	*Paraburkholderia fungorum*, *Escherichia coli*, *Roseomonas gilardii*, *Kocuria indica*, *Staphylococcus epidermidis*	Respiratory infection

a SAP, Severe acute pancreatitis; AP, acute pancreatitis.

**Table 2:** Discordant Culture and mNGS results.

Patient ID	Sample ID	Gender	Disease^a^	Age	Admission date	Discharge date	Sampling date	Bottle positives	Time to positivity	Results of blood culture	Results of plasma mNGS (reads)	Source of blood culture
P03	B03	male	SAP	32	2023/2/18	2023/5/17	2023/4/26	1/4	10	*Acinetobacter baumannii*	Human betaherpesvirus 5	Pancreatic infection
P04	B12	male	SAP	53	2023/3/24	2023/5/11	2023/5/8	1/4, 4/4	26	*Candida auris, Achromobacter xylosoxidans*	*Achromobacter xylosoxidans*, Human betaherpesvirus 5, *Pseudomonas aeruginosa*, *Serratia marcescens*, *Bordetella hinzii*, *Stenotrophomonas maltophilia*, *Klebsiella pneumoniae*, *Candida parapsilosis*	Catheter-related bloodstream infection
P06	B06	male	Glomerulonephritis	67	2023/4/27	2023/5/11	2023/5/3	4/4	4	*Staphylococcus aureus*	Human betaherpesvirus 5, *Kocuria rosea*, *Janibacter melonis*	Catheter-related bloodstream infection
P07	B07	male	AP	36	2023/4/14	2023/5/12	2023/5/4	1/4	39	*Staphylococcus aureus*	Human betaherpesvirus 5, *Klebsiella aerogenes*, *Kocuria palustris*	Intra-abdominal infection
P09	B33	male	SAP	38	2023/3/27	2023/5/19	2023/5/4	1/4	13	*Enterococcus*	Human betaherpesvirus 5	Catheter-related bloodstream infection
P09	B10	male	SAP	38	2023/3/27	2023/5/19	2023/5/6	4/4, 2/4	6, 10	*Enterococcus faecium, Stenotrophomonas maltophilia*	*Lautropia mirabilis*, Human betaherpesvirus 5, *Prevotella intermedia*, *Stenotrophomonas maltophilia*, *Bacteroides fragilis*	Catheter-related bloodstream infection
P11	B13	male	Gastrointestinal hemorrhage	74	2023/5/5	2023/5/10	2023/5/9	2/4	12	*Candida parapsilosis*	*Pseudomonas aeruginosa*, Human betaherpesvirus 5	Respiratory infection
P17	B19	male	SAP	37	2023/5/11	2023/5/22	2023/5/12	2/4, 1/4	35, 54	*Candida parapsilosis, Enterococcus*	*Klebsiella pneumoniae*, *Enterococcus faecium*, Human alphaherpesvirus 2, Human betaherpesvirus 5, Human alphaherpesvirus 1	Pancreatic infection
P17	B20	male	SAP	37	2023/5/11	2023/5/22	2023/5/14	2/4, 1/4	20, 43	*Enterococcus faecium, Candida parapsilosis*	*Gordonia bronchialis*, *Enterococcus faecium*, *Klebsiella pneumoniae*, Human alphaherpesvirus 2, Human betaherpesvirus 5, Human alphaherpesvirus 1	Pancreatic infection
P18	B21	male	SAP	33	2023/5/5	2023/5/28	2023/5/14	4/4	1	*Acinetobacter baumannii*	*Acinetobacter junii*, Human betaherpesvirus 5, Human alphaherpesvirus 1	Catheter-related bloodstream infection
P21	B25	female	SAP	51	2023/5/15	2023/6/19	2023/5/17	4/4	24	*Burkholderia cepacia*	*Staphylococcus epidermidis*, *Elizabethkingia miricola*, Human betaherpesvirus 5	Respiratory infection
P23	B27	male	Chronic renal insufficiency	44	2023/4/25	2023/5/8	2023/4/25	1/4	17	Staphylococcus haemolyticus	*Staphylococcus epidermidis*, Human alphaherpesvirus 2	Contaminant
P24	B28	male	AP	38	2023/4/23	2023/5/5	2023/4/27	1/4	28	Staphylococcus hominis	*Klebsiella pneumoniae*, *Enterococcus faecium*, Human alphaherpesvirus 2, Hepatitis B virus, Human betaherpesvirus, Torque teno virus, Human alphaherpesvirus 2	Catheter-related bloodstream infection
P24	B31	male	AP	38	2023/4/23	2023/5/5	2023/4/28	1/4	25	Staphylococcus hominis	*Klebsiella pneumoniae*, Hepatitis B virus, Torque teno virus	Catheter-related bloodstream infection
P26	B32	female	Crohn’s disease	68	2023/4/27	2023/5/26	2023/5/3	1/4	46	Enterococcus	*Elizabethkingia anopheles*, *Kocuria palustris*, *Klebsiella pneumoniae*, *Candida albicans*	Contaminant
P30	B43	male	SAP	47	2023/5/9	2023/6/2	2023/5/17	2/4	21	Staphylococcus hominis	*Klebsiella pneumoniae*, Human betaherpesvirus 5, Human alphaherpesvirus 1, Human alphaherpesvirus 2, Human gammaherpesvirus 4	Contaminant
P31	B39	female	AP	38	2023/3/8	2023/5/16	2023/5/11	1/4	25	Staphylococcus epidermidis	Staphylococcus capitis, Staphylococcus caprae, Staphylococcus pettenkoferi	Contaminant

a SAP, Severe acute pancreatitis; AP, acute pancreatitis.

### 3.5 Clinical performance of plasma mNGS

We evaluated the clinical performance of plasma mNGS for bacterial and fungal detection using blood cultures as a reference method. Of the 43 samples, plasma mNGS was considered positive only when all pathogens detected by blood culture were detected. The sensitivity and specificity of plasma mNGS were 62.07% (95% CI: 42% to 79%) and 57.14% (95% CI: 29% to 82%), respectively, and the agreement between the two methods was 60% (**Table 3**). Plasma mNGS detected two cases of HBV, nine cases of HSV1, 16 cases of HSV2, 24 cases of CMV, two cases of human gammaherpesvirus 4, and 11 cases of TTV among the 43 samples. Since TTV is also widely present in healthy individuals, and the results of real-time PCR showed that TTV DNA was positive in all 43 samples, TTV was excluded. Using the results of real-time PCR as a reference method, the sensitivity and specificity of plasma mNGS for virus detection were 66.67%–85.71% and 58.62%–100%, respectively (**Table 3**).

**Table 3:** The agreement of plasma mNGS results versus those of blood culture and real-time PCR virus test.

		Plasma mNGS								
		Positive	Negative	Sensitivity (%)	Specificity (%)	PPV (%)	NPV (%)	*p*-value	Kappa	Agreement
Blood culture	Positive	18	11	62.07	57.14	75.00	42.11	0.33	0.18	0.60
	Negative	6	8							
CMV real-time PCR	Positive	12	2	85.71	58.62	50.00	89.47	< 0.05	0.37	0.67
	Negative	12	17							
EBV real-time PCR	Positive	2	1	66.67	100.00	100.00	97.56	< 0.05	0.79	0.98
	Negative	0	40							
HSV1 real-time PCR	Positive	9	3	75.00	96.77	90.00	90.91	< 0.05	0.76	0.91
	Negative	1	30							
HSV2 real-time PCR	Positive	16	5	76.19	90.91	88.89	80.00	< 0.05	0.67	0.84
	Negative	2	20							

CMV, human betaherpesvirus 5; EBV, human gammaherpesvirus; HSV1, human alphaherpesvirus 1; HSV2, human alphaherpesvirus 2.

## 4 Discussion

In this study, we carried out a retrospective study comparing 16S rRNA NGS and mNGS using whole blood and plasma as testing samples for detecting pathogens causing BSIs and compared the results with those of blood culture and viral real-time PCR. We found that, at a fixed depth, the proportion of host reads in whole blood samples exceeded 99%, making it ineffective for the detection and identification of pathogens in patients with sepsis. Compared with 16S rRNA NGS, mNGS could detect more potentially pathogenic bacteria in BSIs. Additionally, a novel finding was that mNGS had a higher detection consistency with blood culture for BSIs caused by gram-negative bacteria, whereas the detection consistency results for catheter-related BSIs caused by gram-positive bacteria was low.

We tested 43 pairs of blood culture-positive samples, with a mean of 64 million (range: 34–70 million) reads. Using whole blood and plasma as testing samples for mNGS, the proportions of host reads were 0.05% and 3.04%, respectively. Internal controls were added to all samples; however, owing to the influence of host DNA, no internal control reads were detected in any of the three samples. The presence of host DNA is unfavorable for the detection and identification of pathogenic microorganisms that cause BSIs (Blauwkamp et al., 2019; Gu et al., 2019; Gu et al., 2021). Although the sequencing depth can be increased, this can significantly increase sequencing costs and the difficulty of data analysis. Using plasma as a testing sample for the metagenomic detection of pathogenic microorganisms causing BSIs is an effective way to reduce detection costs.

While several studies have demonstrated that combining 16S rRNA NGS with blood culture can enhance the sensitivity and specificity of detecting bloodstream infections (BSIs), it is worth noting that in some cases, whole blood and plasma samples were directly used without prior blood culture (El Gawhary et al., 2016; Lelouvier et al., 2016; Moore et al., 2016; Païssé et al., 2016; Rutanga et al., 2018; Fida et al., 2021). Using 16S rRNA NGS with whole blood and plasma as testing samples, 15 and 16 bacteria were detected, respectively, with 10 bacteria identical between the samples and accounting for 67% and 63% of the detections, respectively. This may be because fragmented pathogenic bacterial DNA exists in plasma, whereas intact bacteria may exist in whole blood, resulting in a few differences in the types of bacteria present in the two types of samples. Furthermore, the sensitivity and specificity of 16S rRNA NGS can be significantly impacted by the low pathogenic bacterial load, which can be as low as 1-10 colony-forming units per microliter in whole blood. To improve the detection of BSIs, it is beneficial to use a larger volume of blood (Rutanga et al., 2018). Compared with 16S rRNA NGS for BSIs, plasma mNGS can detect more potentially pathogenic microorganisms, including viruses and fungi (Rodriguez et al., 2020). 16S rRNA NGS is a high-throughput sequencing method targeting the 16S rRNA gene. The copy number of the 16S rRNA gene in plasma or whole blood samples is much lower than that of fragmented pathogenic bacterial DNA. Furthermore, the 16S rRNA gene is not effective in distinguishing certain bacteria (Muhamad Rizal et al., 2020; Stebner et al., 2021; Sun et al., 2023), such as Enterobacteriaceae, *Staphylococcus*. In conclusion, 16S rRNA NGS is not suitable for detecting pathogenic bacteria that cause BSIs.

We screened positive blood culture samples to evaluate the clinical performance of mNGS. The results showed that the sensitivity and specificity of plasma mNGS were 62.07% and 57.14%, respectively, which were lower than those reported in previous studies (Blauwkamp et al., 2019; Wang et al., 2021; Xu et al., 2023). This may be because the proportion of true-positive blood cultures was 67.44% (29/43), which is higher than that reported in previous studies (Hogan et al., 2021; Wang et al., 2021; Falabello De Luca et al., 2023), resulting in a lower sensitivity and specificity of detection. The sources of BSIs were classified, and it was found that the detection rate of gram-negative bacteria, mostly originating from gastrointestinal infections, was higher than that of gram-positive cocci causing catheter-related infections. It has been proposed that the gram-positive bacteria responsible for catheter-related BSIs may have a relatively low abundance. In fact, the average bacterial reads detected in peripheral blood samples were found to be approximately 1/200th of the reads observed in the catheter tip (Yan et al., 2023). When conducting mNGS to detect BSIs, analyzing the potential sources of infection could be more effective, especially for catheter-related infections. This approach can lead to a faster and more accurate diagnosis, allowing timely and appropriate treatment interventions to reduce the burden on patients. No RNA viruses were detected; however, multiple DNA viruses, including CMV, EBV, and HSV, were detected in these patients. These viruses proliferate easily in immunocompromised patients, thereby affecting their prognosis. The positivity rate of CMV detection with plasma mNGS was higher than that with real-time PCR, which may be due to the mismatch of primers and probes for CMV, resulting in the omission of some subtypes. Alternatively, it may be due to the low copy number of the amplification region in real-time PCR, which may have affected CMV detection.

This study has some limitations. First, the sample size was not particularly large, especially regarding the variety of sources of BSIs in patients. Second, the results of blood cultures for some samples may have been affected by sampling issues, leading to some positive blood cultures being considered contaminants, thus affecting our comparative analysis of a large number of positive results. Finally, some mNGS results were not effectively validated, and whether the free DNA of pathogenic microorganisms in the plasma causes BSIs, or is released into the bloodstream from the site of infection after the death of the pathogenic microorganisms remains unclear.

In conclusion, we found that using mNGS with plasma samples is more suitable for detecting BSIs, is less affected by host DNA, and can detect more potential pathogens than 16S rRNA NGS. More importantly, the detection of BSIs caused by gram-negative bacteria was more consistent with blood cultures than with those caused by gram-positive bacteria.

## Data availability statement

The data presented in the study are deposited in the repository of National Genomics Data Center of China (http://ngdc.cncb.ac.cn), with the accession number of PRJCA021184 (https://ngdc.cncb.ac.cn/gsa/s/Am54C9dr).

## Ethics statement

The studies involving humans were approved by the ethical standards of Jinling Hospital of China. The studies were conducted in accordance with the local legislation and institutional requirements. Written informed consent for participation was not required from the participants or the participants’ legal guardians/next of kin because patient information was anonymized and the samples were residual after routine testing.

## Author contributions

JY: Writing – original draft, Conceptualization, Formal Analysis, Investigation, Methodology, Resources. LZ: Conceptualization, Formal Analysis, Resources, Funding acquisition, Writing – original draft. DG: Formal Analysis, Resources, Investigation, Methodology, Writing – original draft. JW: Investigation, Methodology, Writing – original draft. YL: Investigation, Methodology, Writing – original draft. NS: Writing – original draft, Writing – review & editing.
